# Lock and Key: Why *Rickettsia* Endosymbionts Do Not Harm Vertebrate Hosts?

**DOI:** 10.3390/pathogens11121494

**Published:** 2022-12-08

**Authors:** Alejandro Cabezas-Cruz, Andréa Cristina Fogaça

**Affiliations:** 1 ANSES, INRAE, Ecole Nationale Vétérinaire d’Alfort, UMR BIPAR, Laboratoire de Santé Animale, F-94700 Maisons-Alfort, France; 2 Department of Parasitology, Institute of Biomedical Sciences, University of São Paulo, São Paulo 05508-000, SP, Brazil

Are tick endosymbionts transmitted to and able to injure vertebrate hosts? This is a long-standing question concerning both infectiology and microbiology, as microbes (parasites, mutualists, or commensals) shift back and forth from parasitic to mutualistic lifestyles in an evolutionary process dubbed “transitions along the parasite–mutualist continuum” by Drew et al. [1]. Tick-associated microbes are not an exception to this phenomenon [2]. Notable examples include the evolutionary transitions that led a pathogenic strain of *Francisella* to evolve into a *Francisella*-like endosymbiont in the Gulf Coast tick *Amblyomma maculatum* [3], or that caused a maternally inherited *Coxiella*-like endosymbiont to become the Q fever pathogen, *Coxiella burnetii* [4].

The changes in microbial lifestyle involve genomic rearrangements, for instance, size alterations, as indicated for *Coxiella* [4], *Francisella* [3], and *Rickettsia* [5,6,7], among other tick-borne microbes [8]. In general, during their evolutionary transitions from a parasitic to a mutualistic lifestyle, bacterial genomes undergo size reduction [5] and loss in coding capacity [3] but retain their extensive metabolic capabilities [3,4,9]. The latter is an essential trait defining entrance into the “*evolutionary rabbit hole*” of heritable symbiosis [10]. Although carrying commensal/mutualist microorganisms may impose an energetic burden on the host [10], they can also benefit the host, for instance, by producing essential vitamins and conferring resistance to tick-borne pathogens [2].

It was previously reported that the tick intra-mitochondrial symbiont *Midichloria* is present in both tick salivary glands and saliva [11]. Remarkably, bacterial DNA and anti-*Midichloria* antibodies were detected in vertebrate hosts after they were infested by ticks [12], showing that tick endosymbionts can be transmitted to vertebrate hosts. Less clear, however, are some other questions, namely can tick endosymbionts cause disease in vertebrate hosts? What are the lock and key mechanisms to bacteria–host cell interactions that enable a tick-borne microbe to cause disease? A research paper that tackles these questions was published by Kristof et al. [13]. 

In their work, Kristof et al. [13] remarkably showed that certain bacteria in the *Rickettsia* genus recognized as human pathogens (i.e., *R. rickettsii R. parkeri*, *R. africae*, and *R. akari*) can grow within both endothelial and differentiated THP-1 cells (a human leukemia monocytic cell line used as a human macrophage model). In contrast to pathogenic rickettsiae, *Rickettsia bellii*, a tick endosymbiont [6], and the avirulent Iowa strain of *R. rickettsii* can invade both cell types but proliferate only within endothelial cells. They further found that pathogenic rickettsiae grow within THP-1 cytosol but are not localized in the lysosomal compartments, while *R. bellii* was found to be associated with lysosomal compartments. Macrophage xenophagy, the process of the selective capture and degradation of intracellular bacteria by lysosomes, is an essential mechanism of innate immunity to kill and clear infectious bacterial agents [14]. In addition, cell surface recognition and the cytosolic sensing of pathogen-associated molecular patterns (PAMPs) and pathogen-induced damage-associated molecular patterns (DAMPs) result in signaling cascades that promote autophagosome formation and subsequent bacterial targeting for lysosome degradation [14]. In this context, these findings by Kristof et al. [13] are relevant to understand the evolutionary transitions within *Rickettsia*.

The reconstruction of the evolutionary history of *Rickettsia* using genomic comparisons suggested that its ancestor initiated intracellular parasitism in unicellular eukaryotic-like amoebae [5], a trait still conserved in *R. bellii* [6]. Later, it adapted to multicellular eukaryotes [7], first as arthropod symbionts and subsequently as a vector-borne pathogen that affects vertebrates. *Rickettsia bellii* is known to be (*i*) the earliest diverging species of bacteria in the genus *Rickettsia*, (*ii*) the most common rickettsia found in ticks in the American continent, (*iii*) a transovarially transmitted bacterium, and (*iv*) the sole rickettsia found in both soft and hard ticks [6]. The results by Kristof et al. [13] suggest that the tick endosymbiont *R. bellii* may lack or have lost or not acquired one of the essential features of its pathogenic relatives: the mastery to hijack and manipulate the machinery of defensive cells in their vertebrate hosts [15]. Intriguingly, *R. bellii* is able to cause disease in vertebrates, as experimentally proven in guinea pigs [6,16] and rabbits [6], suggesting that it is capable of somehow crossing this apparent “barrier” to infection. Indeed, the intradermal injection of low infectious doses of *R. bellii* is sufficient to cause inflammatory reactions, while high infectious doses induce a black necrotic eschar [6], which is a typical clinical sign of rickettsial pathogenicity [17]. However, notably, the severity of the disease triggered by presumed endosymbionts, including *R. bellii*, but also *Rickettsia amblyommatis* and *Rickettsia montanensis*, is significantly milder than those of recognized rickettsial pathogens such as *R. rickettsii* [16]. Other findings suggested the natural infection of dogs by *R. bellii* [18] and humans by *R. montanensis* and *R. amblyommatis* [19]. 

It was previously shown that the highly lethal *R. rickettsii* is capable of inhibiting apoptosis in host endothelial cells [20,21,22] and tick cells [23]. Indeed, apoptosis inhibition promotes rickettsial growth in tick cells [23]. On the other hand, *R. parkeri*, the causative agent of a mild form of spotted fever, activates apoptosis in tick cells, which also seems to favor infection [24]. Hence, additional comparative studies on the molecular interactions among bacteria in the genus *Rickettsia* and their host/vector cells, such as that reported by Kristof et al. [13], are warranted and may reveal further mechanisms that define the pathogenicity level of rickettsiae. Regardless of the answers to all of the questions presented above, *Rickettsia* spp., independent of their current status in the transitions along “the parasite–mutualist continuum”, should be considered as potential emerging pathogens posing a potential risk to human and animal health.
